# Pharmacognostical Sources of Popular Medicine To Treat Alzheimer’s Disease

**DOI:** 10.2174/1874104501812010023

**Published:** 2018-02-16

**Authors:** Huba Kalász, Shreesh Ojha, Kornélia Tekes, Éva Szőke, Rajesh Mohanraj, Mohamed Fahim, Ernest Adeghate, Abdu Adem

**Affiliations:** 1 Department of Pharmacology and Pharmacotherapy, Semmelweis University, Nagyvárad tér 4, Budapest, Hungary; 2 Department of Pharmacology and Therapeutics, College of Medicine and Health Sciences, United Arab Emirates University, P.O.Box 17666, Al-Ain, United Arab Emirates; 3 Department of Pharmacodynamics, Semmelweis University, 1089 Budapest, Nagyvárad tér 4, Hungary; 4 Department of Pharmacognosy, Semmelweis University, 1085 Budapest, Üllői út 26, Hungary; 5 Department of Physiology, College of Medicine and Health Sciences, United Arab Emirates University; 6 Department of Pharmacology and Therapeutics, College of Medicine and Health Sciences, United Arab Emirates University, P.O.Box 17666, Al-Ain, United Arab Emirates

**Keywords:** Alzheimer’s disease, Anti-AD effect, Etiopathogenesis, Amyloid fibers, Medicinal plants, Galantamine

## Abstract

**Background:**

A large number of classical and recently discovered plants are indicated in preventing and/or treating Alzheimer’s disease (AD).

**Objective:**

Name of plants with their anti-AD effects are important for their further use and investigation.

**Method:**

A short overview of AD is given; anti-Alzheimer plants are given in a Table.

**Results:**

Various medicinal plants are listed here as sources of popular medicines to be used in cases when patients are afraid of developing and/or suffer from AD. Some of these plants have been used for centuries. The major sources in the literature, over one hundred of references are given for plants that show beneficial effect on the progress of AD.

**Conclusion:**

Plant extracts are widely used addition to the synthetic drugs approved by various administrative authorities to stop/slow down the progress of symptoms of AD.

## 1 INTRODUCTION

Currently, Alzheimer’s disease (AD) is one of the leading, neuro-degenerative diseases most commonly affecting people over 65 [1]. Its etiopathogenesis is still shrouded in mystery. Since the description of AD, several landmark discoveries pertaining to the pathogenesis of AD have been postulated. The major findings include the definition of hallmark events such as formation and accumulation of amyloid fibers, and over the years, it has become evident that defective protein processing is involved in AD [2]. The earlier notion was that the onset of AD could present itself years before the establishment of diagnosis, while unambiguous pathological events such as protein misfolding/aggregation could occur at later stages of the progress of the disease. However, after several landmark studies, numerous pathomechanisms have been postulated concerning the development of AD, besides the identification its biomarkers [3]. It is now evident that vascular and metabolic syndromes/dysfunctions, could also play part in AD. In particular, inflammation, oxidative stress and endothelial dysfunction are now believed to promote the development of AD. The involvement of metabolic perturbations in the development of AD was braced by clinical epidemiological studies [4] and was proved to associate with diseases such as diabetes mellitus, dyslipidemia, hypertension, obesity, wherein oxidative stress and insulin resistance were enhanced [5]. Given the complex interplay of cellular signaling and nexus with metabolic diseases, several efforts have been directed to the identification of therapeutic targets/approach to combat AD. Genetic, pharmacological and cell biological studies continue to unravel the possibility of targeting the effector molecules, pathways of inflammation and oxidative stress. Plants have been confirmed to be essential agents in maintaining general well-being for everyone and their importance in the human diet has been unquestioned in modern science [6]. Furthermore, medicinal plants provide a myriad of biologically active molecules and these have been utilized in medicine for thousands of years. The utilization of these special plants and herbs marked the dawn of discovery in the area of pharmacologically active ligands [7]. Neurodegenerative disorders like AD itself are currently a therapeutic enigma with huge socioeconomic impact on improved life expectancy due to advancement in health care. Although the pathological and clinical aspects of these incapacitating disorders are explored to a certain extent, still the treatments applied these days provide only symptomatic relief with no improvement concerning the course or delay in the progression of the disease in question.

An increasing number of herbal extracts, poly-herbal and herbo-mineral preparations and phytochemicals obtained from herbs have been studied for their neuroprotective potential in AD for the past decade. These above mentioned studies have supplied us with utilizable new drug therapies based on medicinal plants.

## 2 METHODS

This review gives an overview of beneficial *in vitro* and *in vivo* adaptation of various plant extracts in AD. The therapeutic potential and postulated ways of action of these agents have been thoroughly examined in multiple experiments in earlier reviews [8, 9]. Recent reviews have focused on the therapeutic potential of traditional medicinal plants and their active components obtained from Chinese herbs for AD and cognitive impairment [10]. As neurofibrillary tangles and amyloid plaques are characterized by deficiency in cholinergic transmission in the basal forebrain, the inhibitors of the enzyme involved in the synthesis of acetylcholine (ACh). The first clinically approved and available AChE inhibitor was tacrine, however, from 2013 onwards it was discontinued in the USA. Nowadays, donepezil and rivastigmine are used in the treatment of AD. Recently, galantamine, an alkaloid obtained from plants of the Amaryllis family, has been developed. Currently, there are still ongoing clinical trials for galantamine. However, approved by the FDA, it is already used for the treatment of mild to moderate AD.

Encouraged by the success of galantamine, several medicinal plants and phytochemicals have been evaluated for the inhibitory effect against acetylcholine esterase enzyme (AChE) in both *in vitro* and *in vivo* models of AD.

A variety of plants reported to show inhibition of AChE, may be considered promising for the treatment of AD associated cognitive decline [11]. Given the clustering of multiple factors involved in the pathogenesis of AD, new therapeutic targets have been selected to develop novel drugs and manage the multiple complications occurring along with AD.

Traditional medicines are being rediscovered for the fulfilment of a strategy aimed at the treatment. Recently, several targets including antioxidants, anti-inflammatory antiapoptotic, neurotrophic and antiamyloid/genesis drugs have been explored in the *in vitro* and *in vivo* models of AD. Naturally occurring dietary polyphenol compounds such as curcumin, resveratrol and green tea catechins have received considerable attention as attractive candidates for AD therapy. These polyphenolic compounds have been demonstrated to prevent AD due to their antioxidant, anti-inflammatory, antiamyloidogenic effects and activating cellular stress adaptive to responses called “neurohormesis” process [12]. The safety and efficacy of herbal medicines as either monotherapy or adjunct to conventional medicines such as cholinesterase inhibitors and nootropic agents for AD have been reviewed based on data from sixteen randomised clinical trials [13]. Fifteen out of the 16 trials provided evidence for the efficacy of herbal medicines in the treatment of AD. *Ginkgo biloba* extract Egb761 has been the most frequently studied. Huperzine A, a cholinesterase inhibitor from *Huperzia serrata* has also been demonstrated to be effective against AD. Based on the systemic review of clinical studies, herbal medicines are believed to be effective and safe medicines for AD treatment.

In this view finding for new compounds effective enough to act on various biochemical targets having new action and low toxicity are requirements hard to meet for both scientists and the pharmaceutical industry. On the other hand, the need to find remedies for this crippling disease has resulted in the reviving interest in natural chemistry. Active constituents of plants, long-known herbs and their extracts may lead to the discovery of medicines successfully used for the treatment of AD. It is generally believed that research on natural product chemistry has a large number of unexploited values capable of providing new possibilities in the treatment of AD.

In this paper, we treat mechanisms that might underlie the improvements in cognitive abilities, neurovascular functions and protection of nerve cells.

## 3 RESULTS AND DISCUSSION

The complete scientific name of the medicinal plants, the possible mechanism(s) of action and relevant references can be found in Table (**1**). A number of medicinal plants, used in Chinese, Ayurvedic, Tibetan, Oriental and Kampo medicines have a reputation enhancing cognitive functions.

Pharmacological investigations of plant-based anti- Alzheimer’s agents have led to the successful introduction of galantamine into the clinical use; clinical trials with or huperzine A are going on.

The degeneration of acetylcholine (ACh) neurotransmission in the central nervous system due to neural degradation is considered to be a major neuropathological characteristic of AD [14]. The animal models of AD are based on the use of scopolamine (SCOP) to block muscarinic ACh receptors, which results in cognitive impairment [15, 16]. The SCOP model is widely used to assess the activity of antidementia agents. At the same time, other neurotoxins, *e.g.* an inhibitor of RNA-protein translation (cycloheximide), a serotonin neurotoxin (para-chloro-amphetamine), the amyloid β-peptide, the ethanol, the aluminum applied on animal models of AD have also proved used to impair cognitive functions. The cognitive or antidementia effects of herbal agents on behavioral pharmacological models, as soon as the passive avoidance test, the Morris water maze test, the T-maze, and both the radial maze paradigms were also checked. Although the mechanisms responsible for the cognition-enhancing effects of most herbal extracts and phytoconstituents are still not completely understood. One or more of the following factors are involved in the improvement of central ACh function; Inhibition of acetylcholinesterase (AChE) and facilitation of ACh synthesis or facilitation of the ACh receptor function; prevention of β-amyloidogenesis by antioxidant action; protection against neurotoxicity and death of nerve cells; facilitation of some neurotrophic effects, *e.g.*, nerve growth factor (NGF). In the field of drug discovery, more attention has been focused on neuroprotection in AD utilizing traditional medicinal plants. Natural compounds with anti- oxidant, anti-inflammatory, anti-apoptotic, and neurofunctional regulational effects may exhibit preventive or therapeutic features against AD. Herbal drugs of traditional medicine which have been well characterized and studied for their behavioral effects and pharmacological properties, may be attractive candidates for further approved for the treatment of patients with Alzheimer's disease, many herbal agents appeared to inhibit of AChE activity.

Tacrine was discriminated in 2013 because of concerns of hepatotoxicity. At the same time, substantial number of herbal extracts and isolated constituents have antioxidant and neuroprotective effects. Antidementia effects are evidenced from **in vitro** results when protection from nerve cell death was induced by exposure to abundant free radicals, excitatory toxins, toxic derivatives of amyloid precursor protein, and other neurotoxins.

## CONCLUSION

Here we reiterate the fact that natural products represent sources of compounds having potential therapeutic implication in AD and other cognitive dysfunctions.

Discovery of drugs from medicinal plants has traditionally been a long process and more cumbersome than other ways of development.

Bioassay screening of extract libraries faces significant difficulties and finding of lead compound libraries is a crucial strategy for discovering drugs. Revealing the precise pathogenesis of AD will give a wider insight into new drug discovery and development and will rationalize drug design with better outcomes and therapeutic benefits. Considering the multitarget, multicomponent action strategy of natural products widely used in the traditional medicine in AD, they offer an unexploited source of new and effective therapy. The traditionally applied herbal preparations offer an adjunctive therapy to drugs acting on amyloidogenesis, AChE and cell death are intensively tested in order to offer better therapeutic prospects in the improvement of learning and memory functions of patients suffering from AD. Currently, very few plant-based medicines are accepted for clinical application. The reasons behind these very few approvals seem to be the complex nature of the chemical components in herbal preparations, difficulties in standardization. Identification of the active ingradient(s) of medical herbs would significantly increase acceptance of these medicinal plants in the clinical practice. The information provided in the present review on numerous plant extracts, formulations and phytoconstituents that have preventive and therapeutic effects on AD in animal models can be used to find new drug therapies for AD and related sequels.

In view of detrimental actions of the agents for AD, in particular treatment refractoriness, high recurrence and side effects that occur in the course of long-term treatments, herbal drugs may offer an alternative possibility. In fact, some human studies have confirmed the beneficial effects of herbal medicines in the prevention and treatment of dementia [17-21]. Based on the encouraging results from animal studies and time-tested safety of some of the herbal agents, the use of these agents as an adjunctive therapy along with conventional drugs can be suggested.

The therapeutic effects of most herbal agents reviewed in this paper are still to be confirmed in clinical settings [22].

## Figures and Tables

**Table 1:** Medicinal plants exhibiting protection from Alzheimer’s disease.

No.	**Medicinal Plants, *Name* and (Family)**	**Mechanism of Action**	**References**
1.	*Abies koreana* E.H. Wilson (Pinaceae)	Improves the memory in scopolamine model of AD in mice.	[23]
2.	*Acorus gramineu* Sol. (Acoraceae)	Exerts AChE inhibitory and antioxidant activity. Increases the learning and memory ability in rat model of AD.	[24, 25]
3.	*Avicennia officinalis* L. *(*Acanthaceae)	Exerts AChE inhibitory activity **in vitro**	[26]
4.	*Bacopa monnieri* (L.) Pennel *(*Plantaginaceae)	Improved cognitive function and reduced loss of neurons in animal model of AD. Enhances learning and memory in randomized double blind placebo controlled trial.	[27-32]
5.	*Berberis darwinii* Hook *(*Berberidaceae)	Exerts AChE inhibitory activity ***in vitro*.**	[33]
6.	*Cassia obtusifolia* (L.), Syn.: *Senna obtusifolia* (L.) H.S. Irwin & Barneby *(*Fabaceae)	Attenuates oxidative stress and Ca^+2^ dysregulation in primary hippocampal cultures.	[34]
7.	*Caulis spatholobi* (L.) *(*Fabaceae)	Exerts AChE inhibitory activity	[35-40, 41, 42-45]
8.	*Celastrus paniculatus* Willd. (Celastraceae)	It sharpens memory and improves concentration as well as cognitive function.	[32]
9.	*Centella asiatica* (L.), Urb. (Apiaceae)	Reduces apoptosis and hippocampal Aβ levels **in vitro** and ***in vivo*.** Enhances learning and memory function in mice models of AD. Potential use in the prevention and treatment of beta-amyloid toxicity and AD.	[32, 46-48]
10.	*Cinnamomum zeylanicum*, Blume (Lauraceae)	Inhibits the formation of Aβ oligomers. Reduces Aβ toxicity in neuronal PC12 cells. Reduces Aβ oligomer and improves cognition in mice model of AD.	[49]
11.	*Citrus medica* (L.) (Rutaceae)	Elicits anti-cholinesterase activity.	[50]
12.	*Cocus nucifera* (L.) (Arecaceae)	Reduces deposition of Aβ in cerebral cortex and tau-1 expression in hippocampus. Protect from amyloidosis and taupathy (neurofibrillary targets in brain of ovarictomized rats. Estrogenic activity.	[51, 52, 53, 54]
13.	*Collinsonia candadensis* (L.) (Lamiaceae)	Called horsebalm. Major constituents are carvacol and thymol that crosses blood-brain barrier which are used for AD,	[55]
14.	*Convolvulus pluricaulis* Choisy (Convolvulaceae)	Dose-dependent enhancement of memory was found in mice.	[32]
15.	*Curcuma longa* (L.) (Zingiberaceae)	Statistics indicate definitely (4.4-fold) lower incidence of AD in countries where Curcuma longa is part of daily diet.	[32]
16.	*Danggui-Shaoyao-San* (Apiaceae)	Improve cognitive function in age related memory dusfunction, reduces Aβ25-35 induced neuronal cell death and antiapoptotic effect in PC12 cells, ameliorate Aβ25-35 induced impairment of spatial learning and memory in mice	[56, 57, 58, 59]
17.	*Desmodium gangeticum* (L.) (Fabaceae)	Elicits AChE inhibitory activity. Improves learning and memory in scopolamine and ageing models of AD in mice	[60]
18.	*Epimedium* koreanum (L.) (Berberidaceae)	Exerts AChE inhibitory activity **in vitro**	[61]
19.	*Ganoderma lucidum* Curtis) P. Karst (Ganodermataceae)	Attenuates Aβ induced synaptotoxicity by preserving synaptophysin and inhibits Aβ induced apoptosis and c-JNK phosphorylation	[62, 63]
20.	*Erigon breviscapus* (Vaniot) Hand.-Mazz. (Asteraceae)	Suppresses lipid peroxidation, expression of nACh α-7 protein, β Apeptide in SH-SY5Y cells	[64, 65]
21.	*Hupezia serrata* (Thunb. ex Murray) Trevis (Huperziaceae)	National Institute of Aging has the clinical trial in Phase II of its extract particularly on AD.	
22.	*Lavendula angustifolia* Mill. (Lamiaceae)	Reduces aggression and improves neuropsychiatric behavior in a cross over randomized trial for treating agitated behaviors of demented people in Hong Kong.	[66, 67, 68, 69]
23.	*Lycium barbarum* (L.) (Solanacea)	Protects against the toxicity of fibrillar Aβ_1-42_ and Aβ_25-35_ in rat cortical neurons Exerts antioxidant and antiapoptotic activity	[70, 71, 72]
24.	*Malus domestica* Borkh. (Rosaceae)	Improves learning and memory in and process organized synaptic signaling in open label trial Exerts antioxidant activity in mice model of AD	[73, 74]
25.	*Morus alba L. syn.: Morus atropurpurea* Roxb. (Moraceae)	Augments the antioxidant defense system Improves learning and memory in mice model of AD	[75]
26.	*Murraya koenigii* Sprangel (Rutaceae)	Improves memory and learning in mice models of AD	[76]
27.	*Oldenlandia affinis* Roem. & Schult. (Rubiaceae)	Inhibits β-secretase activity and decreases Aβ production	[78]
28.	*Paeonia suffruticosa* Andrews (Paeoniceae)	Inhibits β-secretase activity and decreases Aβ production	[79, 80]
29.	*Phangnalon saxatile* (L.) Cass. (Asteraceae)	Exhibits antioxidant and acetyl cholinesterase inhibitory activity.	[81, 82]
30.	*Physostigma venenosa* (L.) Balf. (Labiatae)	Its physostigmine content has relevance to cholinergic therapy in Alzheimer’s disease	[83]
31.	*Pinus nigra* J.F. Arnold, *Syn.: Pinus heldreichii* H. Christ (Pinaceae)	Exerts AChE inhibitory activity **in vitro**	[84]
32.	*Prosopis Africana* (Guill. & Perr) Taub. (Fabaceae)	Inhibits β-secretase activity and decreases Aβ production	[78]
33.	*Pterocarpus erinaceus* Poir. (Fabaceae)	Inhibits β-secretase activity and decreases Aβ production	[85, 86]
34.	*Punica granatum* (L.) (Lythraceae)	Reduces accumulation of Aβ42 and amyloid deposition in hippocampus in transgenic mice (APP (SW)/Tg2576) and protected PC12 cells from H_2_O_2_ induced oxidative stress and increases cognitive function, and inhibits cell death by Aβ induced oxidative stress in mice.	[87-91]
35.	*Paeoniae alba* Pall. (Paeoniaceae)	Exerts AChE inhibitory activity	[35-40, 41, 42-45]
36.	*Salvia miltiorrhizae* Bunge (Labiatae)	Exerts AChE inhibitory activity	[35-40, 41, 42-45]
37.	*Rehmannia glutinosa* (Gaertn) Steud, (Orbanchanceae)	Induces the expression of glial cell line derived neurotropic factor (GDNF) in cells and cultured astrocytes	[92, 93, 94, 95], [96, 97, 98]
38.	*Rhizophora x lamarckii* (Hybrid of *Rhizophora apiculata & R. Stylosa* (Rhizophoraceae)	Exerts AChE inhibitory activity **in vitro**	[26]
39.	*Salvia leriifolia* Benth. (Lamiaceae)	Exerts antioxidant, anti-inflammatory and cholinesterase inhibitory activity.	[99, 100]
40.	*Salvia officinalis, (L)* (Lamiaceae)	Protects PC12 cells from neurotoxicity and tau protein hyperphosphorylation. Improves learning and memory in patients of moderate AD in a double blind randmozed placebo controlled multicenter trial.	[101, 102, 103 104, 105]
41.	*Salvia sclareoides* Brot. (Lamiaceae)	Exerts AChE inhibitory activity **in vitro**	[106, 107, 108]
42.	*Sesuvium portulacastrum (L)* (Aizoaceae)	Exerts AChE inhibitory activity **in vitro**	[26]
43.	*Suaeda monica Forsk. Ex J.F. Gmel.* (Chenopodiaceae)	Exerts AChE inhibitory activity **in vitro**	[26]
44.	*Tabernaemontana divaricata* *(L) R.Br. ex Roem. & Schult.* (Apocynaeeae)	Inhibits cortical AChE activity and enhances cortical neuronal activity	[109]
45.	*Thespesia populnea (L)* (Malvaceae)	Exerts inhibition of AChE activity. Improves learning and memory in diazepam and scopolamine models of AD in mice.	[110]
46.	*Trichilia emetic* Vahl. (Meliaceae)	Inhibits β-secretase activity and decreases Aβ production	[85, 86]
47.	*Uncaria rhynchophylla* (Miq.) (Rubiaceae)	Inhibits fibril formation of both Aβ_1-40_ and Aβ_1-42_ **in vitro**	[111], [112, 113, 114, 115]
48.	*Valeriana amurensis* P. Smirn. (Valerianaceae)	Inhibits the expression of β-APP, Aβ_1-40_ and formation of senile plaques decreases. Reduces pro-inflammatory cytokines and cellular fate of cortical and hippocampal neurons in rat model of AD.	[116, 117, 118, 119]
49.	Vitis *amurensis* Rupr. (Vitaceae)	Inhibits neuronal apoptosis and exhibit antioxidant activity in cultures of rat cortical neurons. Improves learning and memory in mice models of AD	[120]
50.	*Withania somnifera* (L) Dunal (Solanaceae)	Semipurified extract of *Withania somnifera* reverses Alzheimer's disease pathology. Nerving tonic, aphrodistic, rejuvenative, antioxidant activity, calming effect, reverses behavioural deficit.	[32, 121-126]
51.	*Zingiber officinalis* Rosc. (Zingeberaceae)	Exerts Aβ aggregating, antioxidant and AChE inhibitory activity.	[127, 128]
